# Incidence rates of surgically treated rhegmatogenous retinal detachment among manual workers, non-manual workers and housewives in Tuscany, Italy

**DOI:** 10.1007/s00420-013-0894-5

**Published:** 2013-07-28

**Authors:** Stefania Curti, David Coggon, Alberto Baldasseroni, Robin M. T. Cooke, Michela Fresina, Emilio C. Campos, Francesco Semeraro, Francesca Zanardi, Andrea Farioli, Francesco S. Violante, Stefano Mattioli

**Affiliations:** 1 Department of Medical and Surgical Sciences, University of Bologna, Bologna, Italy; 2 MRC Lifecourse Epidemiology Unit, University of Southampton, Southampton, UK; 3 Tuscany Regional Centre for Occupational Injuries and Diseases (CeRIMP), Florence, Italy; 4 Ophthalmology Service, Department of Surgery and Anaesthesiology, University of Bologna, Bologna, Italy; 5 Department of Neurological Sciences and Vision, Eye Clinic, University of Brescia, Brescia, Italy; 6 Unità Operativa di Medicina del Lavoro, Policlinico S.Orsola-Malpighi, via Pelagio Palagi 9, 40138 Bologna, Italy

**Keywords:** Manual work, Retinal surgery, Patient discharge, Incidence study

## Abstract

**Purpose:**

Candidate risk factors for idiopathic rhegmatogenous retinal detachment (RRD) include heavy manual handling (requiring Valsalva’s maneuver). We assessed incidence rates of surgically treated idiopathic RRD among manual workers, non-manual workers and housewives resident in Tuscany, Italy.

**Methods:**

We retrieved all hospital discharge records bearing a principal diagnosis corresponding to RRD coupled with retinal surgery for any resident of Tuscany during 1997–2009. After elimination of repeated admissions and patients with coexistent, associated conditions (including recent trauma), subjects aged 25–59 years were classified as manual workers, non-manual workers or housewives. Population data were extracted from the 2001 census.

**Results:**

We identified 1,946 eligible cases (1,142 men). Among men, manual workers experienced a 1.8-fold higher age-standardized rate per 100,000 person-years than non-manual workers [17.4 (95 % confidence interval (CI) 16.1–18.7) vs. 9.8 (95 % CI 8.8–10.8)]. Age-standardized rates among women were 1.9-fold higher for manual workers [11.1 (95 % CI 9.8–12.3)] and 1.7-fold higher for housewives [9.5 (95 % CI 8.3–10.8)] than in non-manual workers [5.7 (95 % CI 4.8–6.6)].

**Conclusions:**

This large population-based study suggests that manual workers are affected by idiopathic RRD requiring surgical treatment more often than non-manual workers. The higher rates of surgically treated RRD experienced by manual workers are in accord with the hypothesis that heavy manual handling may have a causal role.

## Introduction

Retinal detachment (RD) is a serious ophthalmologic event, which can lead to blindness. It occurs when subretinal fluid accumulates in the potential space between the neurosensory retina and the underlying retinal pigment epithelium. Depending on the mechanism of subretinal fluid accumulation, RD has been classified into rhegmatogenous, tractional, exudative or serous, and combined tractional-rhegmatogenous. Rhegmatogenous retinal detachment (RRD) occurs when a tear in the retina leads to fluid accumulation with a separation of the neurosensory retina from the underlying retinal pigment epithelium; this is the most common type of RD (Ghazi and Green 2002).

In European countries, the reported annual incidence of RRD has varied from 6.3 to 18.2 cases per 100,000 person-years (Laatikainen et al. 1985; Tornquist et al. 1987; Algvere et al. 1999; Mowatt et al. 2003; Mitry et al. 2010b; Van de Put et al. 2013). Age is a known risk factor for RRD, incidence being higher in older people (Mowatt et al. 2003; Polkinghorne and Craig 2004). A recent study reported a peak incidence of 52.5 per 100,000 person-years (95 % confidence interval (CI) 29.4–56.8) at 55–59 years of age (Van de Put et al. 2013). A higher incidence in males has also been reported in previous studies with the male-to-female ratio ranging from 1.3:1 to 2.3:1 (Mitry et al. 2010a).

RRD is often preceded by posterior vitreous detachment (PVD)—defined as a separation between the posterior vitreous cortex and the internal limiting membrane of the retina (Johnson 2010). More than 85 % of RRD cases were found to be associated with PVD and related traction tears (Mitry et al. 2011).

Severe myopia is a major risk factor for RRD, and all myopics are at increased risk (The Eye Disease Case–Control Study Group 1993; Mitry et al. 2010a). Other known risk factors include eye surgery (especially for cataracts) and ocular/head trauma (Austin et al. 1990; Li 2003; Mitry et al. 2011). However, little is known about the role either of social class or of work-related risk factors (other than occupational activities which predispose to serious ocular trauma).

A recent case–control study in Italy, which was restricted to myopic subjects, supported the pathophysiologically plausible hypothesis that occupational heavy manual handling requiring Valsalva’s maneuver is a risk factor for surgically treated RD (Mattioli et al. 2008). Independently from manual handling, high body mass index (BMI) also appeared to carry an increased risk (Mattioli et al. 2008). Subsequently, a complementary analysis of non-myopic cases led us to postulate that heavy lifting and high BMI may also be etiologically relevant in the absence of myopia (Mattioli et al. 2009b).

Contrary to these findings, however, a recent analysis of incident RRD in Scotland indicated that the disease was associated with affluence and higher educational attainment, as measured by a geographical index of deprivation (Mitry et al. 2010b). To explore this apparent discrepancy, we compared incidence rates of surgically treated idiopathic RRD among manual workers, non-manual workers and full-time housewives living in Tuscany, Italy.

## Methods

### Setting and study design

Using hospital discharge records and census data, we calculated and compared age- and sex-specific incidence rates of surgically treated idiopathic RRD experienced by manual workers, non-manual workers and full-time housewives in the general population of Tuscany (3.5 million inhabitants), during the period 1997–2009.

All public and private hospitals in Italy are obliged to produce coded discharge records for all treatment episodes (including day cases), and these are then collated in databases according to the patient’s region of residence (irrespective of where the hospital is located). In addition to the standard data collected elsewhere, the discharge records of hospitals within the administrative Region of Tuscany (*Regione Toscana*) include coded information on the patient’s current broad category of employment (see Table 1), allowing them to be classified as manual workers (i.e., anyone whose job involves some form of manual task other than office work), non-manual workers and full-time housewives.

**Table 1:** Distribution of job categories among surgically treated cases of idiopathic RRD (aged 25–59 years) with known current broad category of employment in Tuscany

	Men (*n* = 1,142)	Women (*n* = 804)	Overall (*n* = 1,946)
Non-manual workers	378	179	557
Managers	35	3	38
Self-employed professionals	105	17	122
Entrepreneurs	25	4	29
Clerical workers	207	152	359
Associate professionals	6	3	9
Manual workers	764	313	1,077
Skilled/unskilled manual workers	172	55	227
Service workers	320	193	513
Home-based workers	2	4	6
Self-employed workers	270	61	331
Housewives	–	312	312

For the present study, we abstracted the records of all patients resident in Tuscany with a discharge record issued by any Italian hospital during the study period giving a principal diagnosis of RRD (ICD-9 code 361.0 through 361.07, and 361.9) coupled with retinal surgery (Diagnosis Related Group code 36). We excluded cases of non-rhegmatogenous RD classified as serous (361.2) or “other” (361.8; including tractional, 361.81). However, we retained patients with diabetes, since this condition is not generally thought to be a risk factor for RRD (as distinct from tractional RD or combined tractional-rhegmatogenous RD). Where a patient was hospitalized for RRD more than once during the study period, only the first episode was abstracted. However, we were not able to identify patients with a history of surgically treated RRD prior to the study period. On the basis of the information archived in the hospital discharge records, we excluded RRD that presented after a recent accident or injury, and patients with an earlier history of cataract surgery, or coexisting aphakia. Thus, the main case definition for the study was incident, surgically treated, idiopathic (i.e., non-traumatic, phakic) RRD.

Although in Italy the age ranges for the working population are wider (at the 2001 census about 62,000 workers were aged 75 years or older), for the calculation of rates among Tuscan manual and non-manual workers and housewives, we restricted the study population to subjects aged 25–59 years because of limited numbers of cases in the youngest age groups and large numbers of retired subjects in the oldest age groups. We also excluded members of the armed forces (due to the difficulty in determining whether their work was manual or non-manual); students (due to possible misclassification in the case of students with concurrent occupational exposure); cases with undeclared/unknown employment status (due to treatment outside Tuscany); unemployed or retired subjects (due to lack of information about previous occupational status); people yet to obtain a first job; and patients with “other” (unspecified) job titles. No house husbands were reported among surgically treated cases of RRD in Tuscany.

To obtain population data for the age groups of interest in the study area, including numbers of manual workers, non-manual workers and full-time housewives, we referred to the closest national census, conducted in 2001 by the National Institute of Statistics (ISTAT).

### Statistical analysis

We calculated age- and sex-specific incidence rates (per 100,000 person-years) for manual workers, non-manual workers and housewives, and also overall rates directly standardized according to the Standard European Population proposed by the World Health Organization (Waterhouse et al. 1976). We calculated age-specific rate ratios (RRs) for male and female manual workers and housewives, taking non-manual workers as the reference category. The likelihood ratio statistic was used to test the null hypothesis that the two rates of interest were equal (Kirkwood and Sterne 2003). To test trends in incidence rates across five-year age bands, we used the score test and derived RR estimates for a unit increase in age class (Clayton and Hills 1993). For both rates and RRs, we calculated 95 % CI.

Since the hospital discharge records database did not permit identification of patients in years before the observation period, we carried out a sensitivity analysis in which we excluded the first 2 years of the observation period (i.e., 1997 and 1998) to explore the possibility that the main analysis might have been distorted by the inclusion of some readmissions of prevalent cases.

Stata 11.2 SE (Stata Corporation, Texas, TX, USA) was used for analysis with a significance level of 0.05.

## Results

Data on employment were available for 2,444 (89 %) of 2,753 surgically treated cases of idiopathic RRD among Tuscan residents aged 25–59 years (age exclusions: ≥60 years, *n* = 4,120; <25 years, *n* = 178). Lack of information on employment within the 25–59 year age group was due either to treatment outside Tuscany (*n* = 106) or undeclared/unknown occupation (*n* = 203). After further exclusion of subjects who had already retired (n = 262), students (*n* = 32), military personnel (*n* = 21), people seeking a first job (*n* = 6), unemployed people (*n* = 49) and unspecified (“other”) job titles (*n* = 128), 1,946 cases entered the main analysis.

Table 1 shows the distribution of job categories among surgically treated cases of idiopathic RRD aged 25–59 years with known current broad category of employment.

Overall age-standardized incidence rates of surgically treated idiopathic RRD (per 100,000 person-years) were 13.7 (95 % CI 12.9–14.5) for men and 8.5 (95 % CI 7.9–9.1) for women.

Among men, the age-standardized rates were 17.4 (95 % CI 16.1–18.7) for manual workers and 9.8 (95 % CI 8.8–10.8) for non-manual workers, corresponding to a 1.8-fold excess in the former.

Age-standardized rates among women were 11.1 (95 % CI 9.8–12.3) for manual workers, 9.5 (95 % CI 8.3–10.8) for housewives and 5.7 (95 % CI 4.8–6.6) for non-manual workers. Thus, female manual workers had a 1.9-fold higher rate of surgically treated idiopathic RRD than their non-manual counterparts, and housewives experienced a 1.7-fold excess.

**Fig. 1:**
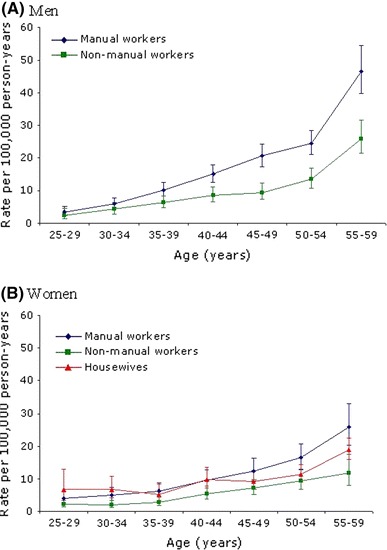
Age-specific incidence rates of surgically treated idiopathic RRD by broad occupational category among men (**a**) and women (**b**) in Tuscany

Figure 1 shows age-specific rates for men and women, according to broad occupational categories (for numbers of cases, see Table 2). Highly significant age-related trends in incidence rates were apparent in all the occupational categories under study: RRs for each 5-year increase in age class were 1.46 (95 % CI 1.41–1.52) for male manual workers, 1.38 (95 % CI 1.31–1.46) for male non-manual workers, 1.36 (95 % CI 1.29–1.45) for female manual workers, 1.38 (95 % CI 1.27–1.50) for female non-manual workers, and 1.22 (95 % CI, 1.15–1.29) for housewives (all *P* < 0.001 in the score test for trend).

**Table 2:** Age- and sex-specific rates (per 100,000 person-years) of surgically treated idiopathic RRD according to broad occupational category in Tuscany

Age (years)	Men						Women								
	Manual workers			Non-manual workers			Manual workers			Non-manual workers			Full-time housewives		
	*n*/*N*	Rate	95 % CI	*n*/*N*	Rate	95 % CI	*n*/*N*	Rate	95 % CI	*n*/*N*	Rate	95 % CI	*n*/*N*	Rate	95 % CI
25–29	28/805,688	3.5	2.4–5.0	11/436,436	2.5	1.4–4.6	20/484,679	4.1	2.7–6.4	12/514,280	2.3	1.3–4.1	9/133,094	6.8	3.5–13.0
30–34	58/970,671	6.0	4.6–7.7	25/578,617	4.3	2.9–6.4	28/555,594	5.0	3.5–7.3	13/639,847	2.0	1.2–3.5	17/252,486	6.7	4.2–10.8
35–39	95/931,879	10.2	8.3–12.5	44/703,261	6.3	4.7–8.4	33/528,866	6.2	4.4–8.8	20/689,884	2.9	1.9–4.5	19/353,301	5.4	3.4–8.4
40–44	120/799,669	15.0	12.5–17.9	56/653,172	8.6	6.6–11.1	45/468,533	9.6	7.2–12.9	33/604,942	5.5	3.9–7.7	36/365,820	9.8	7.1–13.6
45–49	139/676,741	20.5	17.4–24.3	62/653,887	9.5	7.4–12.2	50/404,131	12.4	9.4–16.3	39/547,911	7.1	5.2–9.7	38/415,168	9.2	6.7–12.6
50–54	168/688,220	24.4	21.0–28.4	81/597,584	13.6	10.9–16.9	71/430,937	16.5	13.1–20.8	38/410,345	9.3	6.7–12.7	67/590,460	11.3	8.9–14.4
55–59	156/335,543	46.5	39.7–54.4	99/380,614	26.0	21.4–31.7	66/255,528	25.8	20.3–32.9	24/204,113	11.8	7.9–17.5	126/664,703	19.0	15.9–22.6

Table 3 presents age- and sex-specific RRs for manual workers and (in women only) housewives relative to non-manual workers. These ratios were consistently greater than 1, in most cases to the point of statistical significance.

**Table 3:** Age- and sex-specific RR for manual workers and full-time housewives (with respect to non-manual workers) in Tuscany

Age (years)	Men		Women			
	Manual workers		Manual workers		Housewives	
	RR	95 % CI	RR	95 % CI	RR	95 % CI
25–29	1.4	0.7–2.8	1.8	0.9–3.6	2.9	1.2–6.9^‡^
30–34	1.4	0.9–2.2	2.5	1.3–4.8^†^	3.3	1.6–6.8*
35–39	1.6	1.1–2.3^†^	2.2	1.2–3.8^†^	1.9	1.0–3.5^‡^
40–44	1.8	1.3–2.4*	1.8	1.1–2.8^‡^	1.8	1.1–2.9^‡^
45–49	2.2	1.6–2.9*	1.7	1.1–2.6^†^	1.3	0.8–2.0
50–54	1.8	1.4–2.3*	1.8	1.2–2.6^†^	1.2	0.8–1.8
55–59	1.8	1.4–2.3*	2.2	1.4–3.5*	1.6	1.0–2.5^‡^

* *P* < 0.001; ^†^ *P* < 0.01; ^‡^ *P* < 0.05

A sensitivity analysis excluding the first 2 years of the observation period produced findings very similar to those of the main analysis (data not shown), suggesting that distortion due to inclusion of prevalent cases was unlikely.

## Discussion

This large population-based study indicates that in Tuscany, surgically treated idiopathic RRD is almost twice as common among manual as in non-manual workers. This seems to be in contrast to the association with affluence and higher educational attainment which has been reported from Scotland (Saidkasimova et al. 2009; Mitry et al. 2010b), but consistent with the hypothesis that heavy manual work may be a cause of the disease (Mattioli et al. 2008).

The association with manual work is unlikely to be explained by a confounding effect of myopia, since if anything, myopia tends to be associated with higher levels of education and higher socioeconomic status (Saw et al. 1996). In the EPIC-Norfolk Eye Study, there were no major differences in refractive error between manual and non-manual workers (Foster et al. 2010).

High BMI appears to be associated with surgically treated RD (Mattioli et al. 2008, 2009b) and, even if people of lower socioeconomic status are more likely to have higher BMI (Vannoni et al. 2005), this is unlikely to have caused important confounding since the prevalence of overweight/obese subjects in Tuscany is very low [National Institute of Statistics (ISTAT) 2002].

The apparent discrepancy with findings in Scotland might, however, relate in part to later presentation to hospital in that country by patients with RRD from deprived areas. Thus, Mitry et al. observed that “RRD cases from more deprived datazones frequently present with a more extensive area of detachment” (Mitry et al. 2010b).

It is also possible that residence in a more deprived area is a poor marker for manual work. Many manual workers may live in less deprived areas, and a relatively high proportion of residents from the most deprived areas in Scotland may have been unemployed. This would be relevant if the association that we observed with manual work in our study was a consequence of heavy manual handling. In general, manual workers perform such tasks much more frequently than non-manual workers and the unemployed, who will encounter the exposure mainly outside work when performing domestic tasks or practicing sports and other hobbies. Thus, in the Fifth European Working Conditions Surveys, the proportion of manual workers who reported carrying or moving loads for at least a quarter of their total working time was 47.2 % (95 % CI 43.7–50.8 %) as compared with 7.6 % (95 % CI 5.7–9.5 %) for non-manual workers (European Foundation for the Improvement of Living and Working Conditions 2005).

Among women, we found that in comparison with non-manual workers, rates of surgically treated idiopathic RRD were elevated not only in manual workers, but also in full-time housewives. Possible explanations include an effect of BMI and parity, which in Italy tend to be higher in housewives than in non-manual workers (Mattioli et al. 2009a). Moreover, housewives may also carry out heavy manual handling more often than non-manual workers in the course of their household tasks.

In line with previous studies (Mitry et al. 2010a; Van de Put et al. 2013), our study suggests that surgically treated idiopathic RRD is more frequent among men than women (even among non-manual workers) and increases with age. Our study could not provide information about other known or hypothesized risk factors, due to a lack of such data in the hospital discharge records.

Because all Italian hospitals are required to supply discharge records to local administrations, we were able to ascertain the vast majority of eligible surgically treated cases in the general population. The accuracy of the database is nowadays considered of high quality: in Tuscany, the number of errors in the coding of diagnosis and treatment is 3 and 1.5 per 1,000 records, respectively (Italian Ministry of Health 2011). In our study, the case definition was based on both diagnosis and treatment; hence, the possibility of false positives was very low. However, the data that were available on individual patients were limited, and this precluded adjustment for potential confounders other than age and sex (including myopia and BMI). Moreover, there was no quantification of duration, type or intensity of job tasks and exposures. Furthermore, our attempt to restrict the definition of cases to “idiopathic” RRD may have been compromised by underreporting of concomitant conditions in the discharge records.

The use of denominator data from the 2001 census to calculate rates over a longer time frame (1997–2009) could have biased estimates somewhat. Employment data were not available for other years in the study period, and it was therefore necessary to assume that populations of manual workers, non-manual workers and housewives were fairly constant over time. We are not aware of any major changes in employment patterns in Tuscany during the study period, and this assumption therefore seemed reasonable. Moreover, when we compared the distribution of the general population by age class and gender across the years of study, there were no substantial differences from those in the 2001 census (data not shown). To produce important bias, there would have had to be a large change in patterns of employment over a relatively short period.

We excluded from the analysis 106 patients treated outside Tuscany due to lack of information on employment. It should be noted that about 70 % of those patients attended hospitals in adjacent regions, probably because the hospital in the region concerned was closer than others located in Tuscany. Even if all those patients had been non-manual workers, there would still have been a higher incidence in manual than non-manual workers. Only one-third of the patients not resident in the region, but surgically treated for RRD in Tuscan hospitals, were non-manual workers (data not shown).

Exclusion of retired subjects from the main analysis (due to lack of information on occupational history) limits the extent to which our findings can be generalized. However, if the risks associated with manual work derived only from recent exposure to relevant occupational activities, inclusion of retired subjects might have led to a reduction in the association.

To address possible discrepancies in occupational classification between cases and the general population, we excluded from the analysis occupational groupings that were not readily classifiable into manual or non-manual categories (namely, military personnel and subjects with “other” or unknown occupational status). It is still possible that some misclassification of occupation occurred, although since both the hospital discharge records and census data had coded categories specifically for full-time housewives, misclassification of housewives is not a major concern.

In the absence of data on ethnicity, we do not know to what extent different ethnic groups contributed to the overall incidence rates in the population studied. However, the very low proportion (about 2 %) of non-Italian citizens among the surgically treated cases makes it likely that the overall incidence rates were fairly representative of a native Italian population.

As regards the external validity of the findings, it is noteworthy that the overall age-standardized incidence rates of surgically treated idiopathic RRD were broadly in line with those reported in another population-based study (Wong et al. 1999). However, it is likely that the relative frequencies of surgery in the three occupational categories may have been influenced by the composition of the Tuscan workforce (distribution of manual job titles, etc.).

This work provides further evidence that idiopathic RRD requiring surgical treatment has important social determinants and highlights the scope for identification of personal and occupational risk factors as a necessary step for prevention. Considering that manual workers perform heavy manual handling much more frequently than non-manual workers, the higher rates of RRD experienced by manual workers support the hypothesized causal role of occupational manual handling. This might be explained by various factors related to Valsalva’s maneuver, such as vitreal traction, raised pressure in the choroid and possibly even recurrent Valsalva hemorrhagic retinopathy (Mattioli et al. 2008). If confirmed by further studies with more specific measures of exposure, it would strengthen the case for controls on heavy lifting in the workplace, and it would enable identification of populations at higher risk who might be warned about symptoms of RD and the importance of seeking medical advice early should they occur. 

## References

[CR1] Algvere PV, Jahnberg P, Textorius O (1999). The Swedish Retinal Detachment Register. I. A database for epidemiological and clinical studies. Graefes Arch Clin Exp Ophthalmol.

[CR2] Austin KL, Palmer JR, Seddon JM, Glynn RJ, Rosenberg L, Gragoudas ES, Kaufman DW, Shapiro S (1990). Case-control study of idiopathic retinal detachment. Int J Epidemiol.

[CR3] Clayton D, Hills M (1993). Statistical models in epidemiology.

[CR4] European Foundation for the Improvement of Living and Working Conditions (2005) European Working Conditions Survey. Data Archive (distributor), Colchester, Essex

[CR5] Foster PJ, Broadway DC, Hayat S, Luben R, Dalzell N, Bingham S, Wareham NJ, Khaw KT (2010). Refractive error, axial length and anterior chamber depth of the eye in British adults: the EPIC-Norfolk Eye Study. Br J Ophthalmol.

[CR6] Ghazi NG, Green WR (2002). Pathology and pathogenesis of retinal detachment. Eye (Lond).

[CR7] Italian Ministry of Health (2011) Annual report on hospital discharge records (based on 2010 data). http://www.salute.gov.it/ricoveriOspedalieri/ricoveriOspedalieri.jsp. Accessed 7 January 2013

[CR8] Johnson MW (2010). Posterior vitreous detachment: evolution and complications of its early stages. Am J Ophthalmol.

[CR9] Kirkwood BR, Sterne JAC (2003). Essential medical statistics.

[CR10] Laatikainen L, Tolppanen EM, Harju H (1985). Epidemiology of rhegmatogenous retinal detachment in a Finnish population. Acta Ophthalmol (Copenh).

[CR11] Li X (2003). Incidence and epidemiological characteristics of rhegmatogenous retinal detachment in Beijing, China. Ophthalmology.

[CR12] Mattioli S, De Fazio R, Buiatti E, Truffelli D, Zanardi F, Curti S, Cooke RM, Baldasseroni A, Miglietta B, Bonfiglioli R, Tassinari G, Violante FS (2008). Physical exertion (lifting) and retinal detachment among people with myopia. Epidemiology.

[CR13] Mattioli S, Baldasseroni A, Bovenzi M, Curti S, Cooke RM, Campo G, Barbieri PG, Ghersi R, Broccoli M, Cancellieri MP, Colao AM, Dell’omo M, Fateh-Moghadam P, Franceschini F, Fucksia S, Galli P, Gobba F, Lucchini R, Mandes A, Marras T, Sgarrella C, Borghesi S, Fierro M, Zanardi F, Mancini G, Violante FS (2009). Risk factors for operated carpal tunnel syndrome: a multicenter population-based case-control study. BMC Public Health.

[CR14] Mattioli S, Curti S, De Fazio R, Farioli A, Cooke RM, Zanardi F, Violante FS (2009). Risk factors for retinal detachment. Epidemiology.

[CR15] Mitry D, Charteris DG, Fleck BW, Campbell H, Singh J (2010). The epidemiology of rhegmatogenous retinal detachment: geographical variation and clinical associations. Br J Ophthalmol.

[CR16] Mitry D, Charteris DG, Yorston D, Siddiqui MA, Campbell H, Murphy AL, Fleck BW, Wright AF, Singh J (2010). The epidemiology and socioeconomic associations of retinal detachment in Scotland: a two-year prospective population-based study. Invest Ophthalmol Vis Sci.

[CR17] Mitry D, Singh J, Yorston D, Siddiqui MA, Wright A, Fleck BW, Campbell H, Charteris DG (2011). The predisposing pathology and clinical characteristics in the Scottish retinal detachment study. Ophthalmology.

[CR18] Mowatt L, Shun-Shin G, Price N (2003). Ethnic differences in the demand incidence of retinal detachments in two districts in the West Midlands. Eye (Lond).

[CR19] National Institute of Statistics (ISTAT) (2001) General population data. http://www.istat.it/it/prodotti/banche-dati. Accessed 23 November 2012

[CR20] National Institute of Statistics (ISTAT) (2002) Indagine multiscopo sulle famiglie. Condizioni di salute e ricorso ai servizi sanitari 1999–2000 Roma

[CR21] Polkinghorne PJ, Craig JP (2004). Northern New Zealand Rhegmatogenous Retinal Detachment Study: epidemiology and risk factors. Clin Exp Ophthalmol.

[CR22] Saidkasimova S, Mitry D, Singh J, Yorston D, Charteris DG (2009). Retinal detachment in Scotland is associated with affluence. Br J Ophthalmol.

[CR23] Saw SM, Katz J, Schein OD, Chew SJ, Chan TK (1996). Epidemiology of myopia. Epidemiol Rev.

[CR24] The Eye Disease Case-Control Study Group (1993). Risk factors for idiopathic rhegmatogenous retinal detachment. Am J Epidemiol.

[CR25] Tornquist R, Stenkula S, Tornquist P (1987). Retinal detachment. A study of a population-based patient material in Sweden 1971–1981. I. Epidemiology. Acta Ophthalmol (Copenh).

[CR26] Van de Put MA, Hooymans JM, Los LI (2013). The incidence of rhegmatogenous retinal detachment in the Netherlands. Ophthalmology.

[CR27] Vannoni F, Demaria M, Quarta D, Gargiulo L, Costa G (2005). Differences of perceived health and lifestyle by occupational groups in the Italian ISTAT (Central Statistic Institute) health survey. Med Lav.

[CR28] Waterhouse J, Muir CS, Correa P, Powell J (1976). Cancer incidence in five continents, vol III.

[CR29] Wong TY, Tielsch JM, Schein OD (1999). Racial difference in the incidence of retinal detachment in Singapore. Arch Ophthalmol.

